# Maturation of Murine Bone Marrow-Derived Dendritic Cells Induced by *Radix Glycyrrhizae* Polysaccharide

**DOI:** 10.3390/molecules17066557

**Published:** 2012-05-30

**Authors:** Xiaobing Li, Xiaojuan He, Biao Liu, Li Xu, Cheng Lu, Hongyan Zhao, Xuyan Niu, Shilin Chen, Aiping Lu

**Affiliations:** 1 School of Basic Medicine, Henan University of Traditional Chinese Medicine, Zhengzhou 450008, China; 2 Institute of Basic Research in Clinical Medicine, China Academy of Chinese Medical Sciences, Beijing 100700, China; 3 School of Life Sciences, Hubei University, Wuhan 430062, China; 4 College of Life Science and Technology, Beijing University of Chemical Technology, Beijing 100029, China; 5 Institute of Basic Theory, China Academy of Chinese Medical Sciences, Beijing 100700, China; 6 Institute of Medicinal Plant Development, Chinese Academy of Medical Sciences & Peking Union Medical College, Beijing 100193, China; 7 School of Chinese Medicine, Hong Kong Baptist University, Hong Kong, China

**Keywords:** *Radix Glycyrrhizae* polysaccharide, dendritic cells, maturation, immune regulation

## Abstract

*Radix Glycyrrhizae* polysaccharide (GP), the most important component of *Radix Glycyrrhizae*, has been reported to have many immunopharmacological activities. However, the mechanism by which GP affects dendritic cells (DCs) has not been elucidated. In this study, we investigated the effect of GP on murine bone marrow-derived DCs and the potential pathway through which GP exerts this effect. Mononuclear cells (MNCs) were isolated from murine bone marrow and induced to become DCs by culturing with GM-CSF and IL-4. Six days later, DCs were divided into three groups: control group, GP group and LPS group. After 48 h of treatment, phenotypic figures and antigen uptake ability were determined by FACS analysis. The proliferation of DC-stimulated allogenic CD3+ T cells was detected by WST-1. IL-12 p70 and IFN-γ, which are secreted by DCs and CD3+ T cells respectively, were quantified by ELISA. Additionally, IL-12 p40 mRNA expression was determined by real-time PCR. Alterations in TLR4-related signaling pathways were examined by performing an antibody neutralization experiment. Treatment of DCs with GP resulted in the enhanced expression of the cell surface molecules CD80, CD86 and MHC I-A/I-E. GP also increased the production of IL-12 p70 by DCs in a time-dependent manner. The endocytosis of FITC-dextran by DCs was suppressed by GP administration. Furthermore, GP-treated DCs enhanced both the proliferation and IFN-γ secretion of allogenic CD3+ T cells. Finally, the effects of GP on DCs were partially reduced by using inhibitors of TLR4, NF-κB, p38 MAPK or JNK. In conclusion, GP can induce the maturation of DCs, and does so, in part, by regulating a TLR4-related signaling pathway.

## 1. Introduction

*Glycyrrhiza glabra* is a well-known Chinese herb that has been used in food and medicinal remedies for thousands of years [1]. This herb has long been valued as a demulcent (soothing, coating agent), and serves to relieve respiratory ailments (such as allergies, bronchitis, colds, sore throats and tuberculosis), stomach burn (including heartburn from acid reflux or other causes), gastritis, inflammatory disorders, skin diseases and liver problems [2]. The medicinal and pharmacological uses of *Glycyrrhiza glabra* have been described in several studies [3,4,5]. *Radix Glycyrrhizae* Polysaccharide (GP), one of main bioactive components in *Radix Glycyrrhizae*, has many pharmacological effects. Recently, GP has been reported to participate in multiple processes, including the regulation of immunity [6] and phagocytosis [7], as well as anti-complement [8], anti-viral [9], and anti-tumorigenic [10] processes. Additionally, GP exhibits low cellular toxicity [10]. Prior to this study, the mechanism by which GP exerts its immunoregulatory activity remained unclear. In particular, it was previously unknown whether GP elicited its effects through modulating important immune cells such as dendritic cells (DCs).

DCs are the most potent antigen presenting cells, possessing the unique ability to link innate and adaptive immunity. DCs act as immune sentinels to initiate T cell responses against microbial pathogens, inflammation and tumors [11,12]. The maturation process is central to the function of DCs [13]. DC maturation can be initiated both *in vivo* and *in vitro* by many stimuli, including pro-inflammatory cytokines and bacterial products, such as lipopolysaccharide (LPS) [14,15,16]. Fully mature DCs exhibit an increase in the surface expression of MHC II and costimulatory molecules (such as CD80 and CD86) but a decrease in the capacity to internalize antigens [17]. The induction of DC maturation is critical for the generation of Ag-specific T lymphocyte responses and may be essential for the development of human vaccines that rely on T cell immunity. Additionally, IL-12 production is an important marker of DC maturation and can be used to identify Th1-inducing adjuvants [18]. LPS recognized by TLR4 in DC, the nuclear transcription factor (NF-κB) and the MAPK signaling pathway also play important role in DC maturation [19,20]. Recent reports have identified a number of polysaccharides that participate in the process of DC maturation and induced the MAPK signaling pathway in monocyte-derived DCs [21,22]. The present study is the first to examine the consequence of GP on the maturation of murine bone marrow-derived DCs and to investigate a potential mechanism by which GP exerts it effects.

## 2. Results and Discussion

### 2.1. GP Induces Phenotypic Maturation of DCs

**Figure 1:**
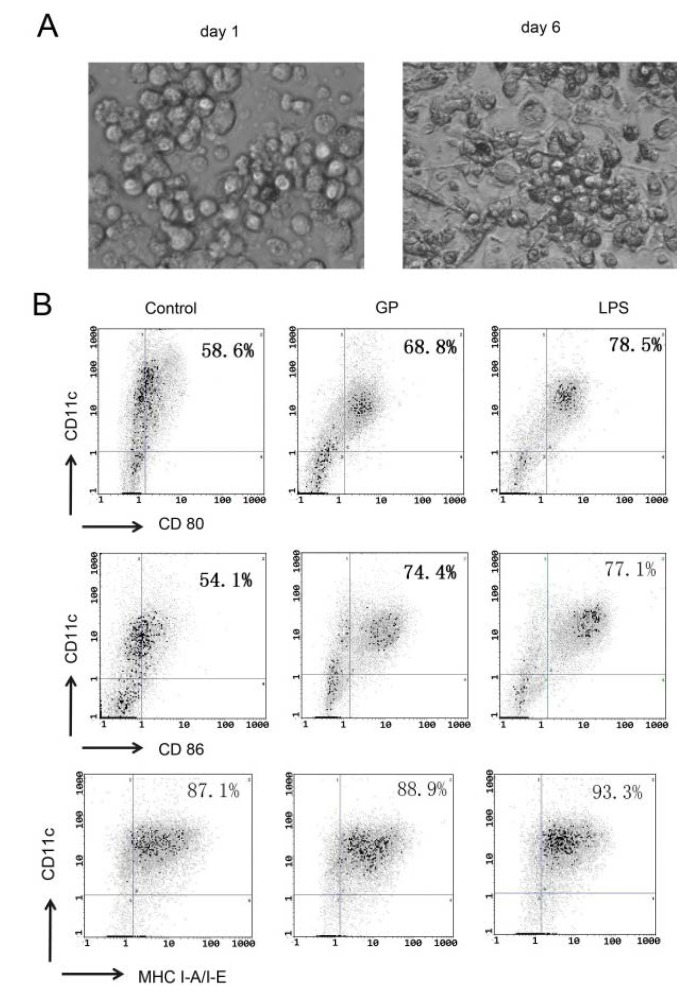
Effects of GP on phenotypic maturation of DCs. Hematopoietic progenitor cells from the bone marrow of C57BL/6 mice were cultured in the presence of GM-CSF and IL-4 for 6 days. Then, day 6 BMDCs were stimulated with GP (100 μg/mL) or LPS (100 ng/mL) for 48 h. Cells were then stained with two Abs, the first Ab was PE-conjugated CD11c, and the second Ab was FITC-CD80, FITC-CD86 or FITC-MHC I-A/I-E. Cells were analyzed by flow cytometry. The results are representative of three independent experiments. (**A**) Morphological characteristics of BMDCs (×200). The left picture is that bone morrow cells induced by GM-CSF and IL-4 for 1 day; the right picture is that bone morrow cells induced by GM-CSF and IL-4 for 6 days; (**B**) Flow cytometry results.

Hematopoietic progenitor cells from the bone marrow of C57BL/6 mice were cultured in the presence of GM-CSF and IL-4 for 6 days to induce DCs. The cells were observed with inverted phase contrast microscope. Figure 1A shows that mature DCs are large cells with oval or irregularly shaped nuclei and abundant cytoplasm. Some cells exhibited the formation of dendrites. Six days later, DCs were stimulated with GP (100 μg/mL) or LPS (100 ng/mL) for 48 h. The expression of CD80, CD86 and MHC I-A/I-E was examined. As shown in Figure 1B, GP increased the expression of CD80, CD86 and MHC I-A/I-E, which are established surface markers used to identify mature DCs.

### 2.2. GP Inhibits the Endocytic Activity of DCs

**Figure 2:**
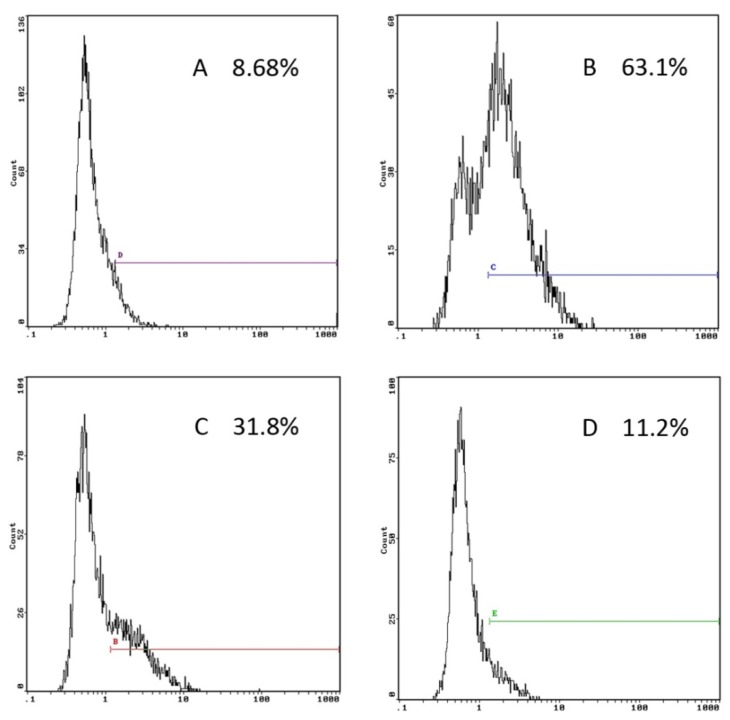
Effects of GP on the endocytotic capacity of DCs. Day 6 BMDCs were stimulated with GP (100 μg/mL) or LPS (100 ng/mL) for 48 h; and then incubated with FITC-dextran for 1 h at 37 °C. As a control, cells were also incubated at 4 °C. (**A**) 4 °C control; (**B**) 37 °C control; (**C**) GP; (**D**) LPS. The results are representative of three independent experiments.

Immature DCs have a high endocytic capacity, but during differentiation they lose their endocytic activity towards antigens and mature into potent immunostimulatory antigen presenting cells (APC) [23]. To assess whether GP has an effect on the endocytic activity of DCs, BMDCs cultured in the presence or absence of GP (100 μg/mL) were incubated with the fluorescent marker dextran-FITC. The results are shown in Figure 2. Compared with the control group, the endocytic activity of GP-treated DCs was significantly decreased. Parallel experiments were performed at 4 °C to examine the non-specific uptake of dextran-FITC into DCs.

### 2.3. GP-Treated DCs Enhance Allogenic T Cell Proliferation

**Figure 3:**
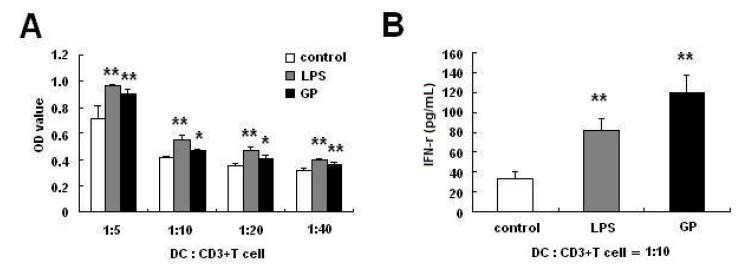
GP-treated DCs strengthen allogenic T cell proliferation and IFN-γ secretion. Day 6 BMDCs were stimulated with LPS (100 ng/mL) or GP (100 µg/mL) for 48 h. Allogeneic CD3+ T cell proliferation was measured after 5 days of co-culture with DCs. DCs were cultured with CD3+ T cells at a ratio of 1:5, 1:10, 1:20 or 1:40. After co-culturing DCs with T cells at a ratio of 1:10 for 48 h, the culture supernatants were collected and IFN-γ was measured. (**A**) GP-treated DCs strengthen allogenic T cell proliferation; (**B**) GP-treated DCs increase IFN-γ secretion of allogeneic T cells. Compared with the control group, * *p* < 0.05, ** *p* < 0.01. The results are representative of three independent experiments.

Mature DCs can activate T cells. We examined the ability of the GP-treated DCs to induce a T-cell response. As shown in Figure 3A, after co-culture for 5 days, the capacity of DCs treated with GP (100 μg/mL) or LPS (100 ng/mL) to induce the proliferation of allogeneic T cells was similar, and it was markedly higher than that of untreated DCs. Furthermore, in order to determine whether GP-treated DCs modulate cytokine secretion of allogeneic T cells, we measured IFN-γ expression in the supernatants after co-culture for 2 days. As shown in Figure 3B, GP-treated DCs significantly increased secretion of the cytokine IFN-γ (*p* < 0.01).

### 2.4. GP Enhances IL-12 Production in DCs

**Figure 4:**
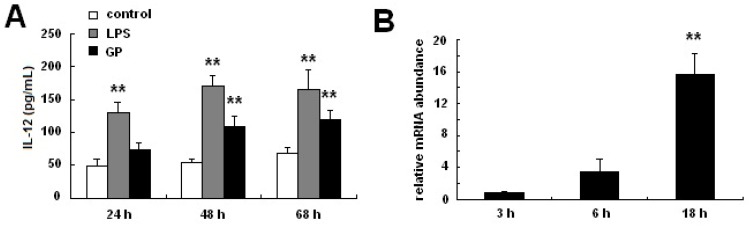
Effects of GP on IL-12 p70 production in DCs. For the IL-12 p70 assay, DCs were established in culture as described in materials and methods. GP was then added to the wells for 48 h, and the culture supernatants were harvested at various times for measurement of IL-12 p70 concentration using ELISA. IL-12 p40 mRNA expression was measured using real-time PCR. (**A**) Concentration of IL-12 p70 produced by GP-treated DCs; (**B**) IL-12 p40 mRNA expression in GP-treated DCs. Compared with the control group, ** *p* < 0.01. The results are representative of three independent experiments.

IL-12, which exists as a disulfide-linked p35/p40 heterodimer, is a functional DC maturation marker [24]. To investigate the effects of GP on IL-12 p70 expression in DCs, BMDCs were incubated with GP (100 μg/mL) for different time. As shown in Figure 4A, GP significantly increased the production of IL-12 p70 in BMDCs at 48 h and 68 h. Additionally, IL-12 p40 mRNA expression was determined by quantitative RT-PCR. As shown in Figure 4B, GP increased IL-12 p40 mRNA expression in DCs in a time-dependent manner.

### 2.5. GP Induces IL-12 p70 Synthesis through TLR4 Signaling Pathways

**Figure 5:**
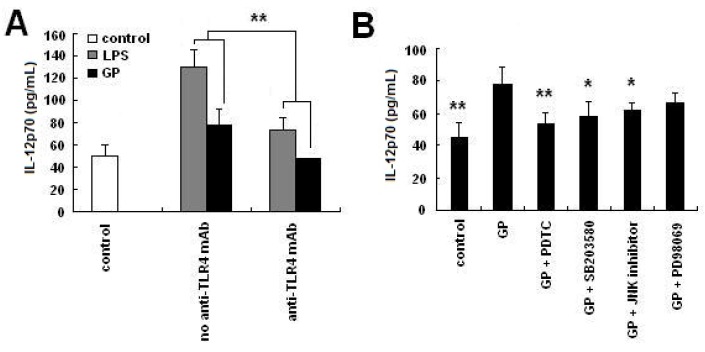
Effects of TLR4-related signaling pathways on IL-12 p70 production in GP-treated DCs. Day 6 BMDCs were pre-treated with inhibitors of TLR4, NF-κB, p38 MAPK, ERK or JNK for 1 h and then incubated with GP for 24 h. Isotype control mAb was added into control group. The supernatant was then collected for IL-12 p70 detection by ELISA. (**A**) Effect of anti-TLR4 mAb on IL-12 p70 production of GP-treated DCs. Compared with the no anti-TLR4 mAb group, ** *p* < 0.01; (**B**) Effect of TLR4 downstream signaling pathways on IL-12 p70 production of GP-treated DCs. Compared with the GP group, * *p* < 0.05, ** *p* < 0.01. The results are representative of three independent experiments.

DCs express surface receptors, including TLR family members, which allow them to recognize pathogen associated molecular patterns (PAMP). The TLR family member TLR4 is involved in the detection of bacterial LPS [25]. To determine whether TLR4 recognized GP, neutralization experiments were performed. Cell surface TLR4 receptors were blocked by the addition of a neutralizing antibody before BMDCs were treated with GP. As shown in Figure 5A, the addition of an anti-TLR4 mAb blocked GP-induced and LPS-induced IL-12 p70 production. Compared with the unblocked group, which did not receive anti-TLR4 mAb, the expression of IL-12 p70 decreased 35.6% in the GP group, and 39.8% in the LPS group. To further investigate the effect of GP on TLR4-downstream signaling pathways, a second blocking experiment was performed. BMDCs were pre-treated with various blocking antibodies for 1 h and subsequently stimulated with GP for 24 h. As shown in Figure 5B, GP induced significant production of IL-12 p70, which was substantially blocked by inhibiting NF-κB, p38 MAPK or JNK. In contrast, ERK inhibition had little effect on the GP-induced production of IL-12 p70.

### 2.6. Discussion

In previous studies, research has focused on the biological effects of GP in macrophages [26,27], as well as the effects of GP on the proportion of Treg cells in tumor-bearing mice [28]. This is the first report to demonstrate that GP can induce the maturation of murine DCs through a mechanism that partially involves a TLR4-related signaling pathway.

DC maturation can be divided into four stages [21,22]: precursor DCs, immature DCs, migration DCs and mature DCs. Among these stages, immature DCs and mature DCs are the main focus of research. Immature DCs exhibit low expression of MHC-II, co-stimulatory molecules and adhesion molecules, while mature DCs exhibit high expression of these molecules. Additionally, immature DCs are particularly efficient at antigen uptake and processing, while mature DCs are characterized by enhanced antigen presenting capacity. Our results demonstrated that, after 8 days in culture, morphologically typical DCs were present and expressed high levels of the cell surface molecules CD80, CD86 and MHC I-A/I-E. GP further enhanced expression of these cell surface molecules and decreased DCs endocytic capacity.

Mature DCs are capable of increasing allogeneic T cell stimulation and producing IL-12 [18], which is a T cell stimulating factor and involved in the differentiation of Th0 cells into Th1 cells [29]. Our results showed that GP could enhance the expression of IL-12 by DCs in a time-dependent manner, and that GP could significantly strengthen the proliferation of CD3+ T cells and initiate Th1 responses. CD3+ T cells stimulated with GP-treated DCs produced elevated levels of IFN-γ. Taken together, these data strongly suggest that GP functions to induce not only phenotypic, but also functional maturation of DCs.

TLR4 detects LPS on Gram-negative bacteria and is thus important in the activation of the innate immune system. TLR4 has been shown to interact with MyD88, a Toll/IL-1 receptor (TIR)-containing intracellular adaptor protein [30,31]. The MyD88-dependant signaling pathway activates JNK, MAPKs and NF-κB. In this study, GP induced significant production of IL-12 p70, which was strongly inhibited by TLR4, NF-κB, p38 MAPK or JNK inhibitors. The results indicated that GP could be recognized at least in part by TLR4, and that the induction of the NF-κB and MAPKs signaling pathways might contribute to the enhancement of DCs maturation. Certainly, it is worthy of further studies to investigate the role of other TLRs and signaling pathways involving in the activities of GP.

## 3. Experimental

### 3.1. Mice

Female C57BL/6 and BALB/c mice (6–8 weeks of age) were obtained from The Military Medical Science Academy of the PLA, China. The animals were housed in a regulated environment (22 ± 1 °C, relative humidity 60 ± 5%) under a 12 h light/dark cycle. All animals were acclimatized for at least one week prior to the experiments. Food and water were administrated *ad libitum*. The approval of the Institutional Animal Ethics Committee was obtained prior to carrying out animal experiments.

### 3.2. GP and Other Chemicals

*Radix Glycyrrhizae* Polysaccharide (GP) was purchased from Pharmagenesis, Inc. (Redwood City, CA, USA). The percentage of polysaccharide of GP was about 93.52%. The molecular weights of GP were estimated to be 80,000 Daltons based on high performance gel filtration chromatography with pullulans as standards. There was no detectable endotoxin (<0.10 endotoxin units/mL) in the GP samples, as deternined by Endospecy. *Escherichia coli* LPS (026:B6, *E. coli*) was purchased from Sigma. (St. Louis, MO, USA).

### 3.3. Induction of Mouse Bone Marrow-Derived DCs

Bone marrow-derived DCs (BMDCs) were induced from bone marrow (BM) cells obtained from 6–8 week-old mice [32]. Briefly, a single cell suspension was prepared from BM obtained from femurs and tibias. After lysing red blood cells, whole BM cells (2 × 10^6^ cells/mL) were cultured in RPMI 1640 medium in six-well flat bottom plates (Orange Scientific, Braine-l’Alleud, Belgium) at 37 °C, 5% CO2, supplemented with 10% fetal calf serum (FCS), 2 mM L-glutamine, 100 U/mL penicillin G, 100 mg/mL streptomycin, 30 ng/mL reconstructive mouse GM-CSF (Peprotech, Rocky Hill, USA) and 20 ng/mL reconstructive mouse IL-4 (Peprotech). Cells were incubated for 24 h. Plates were then gently swirled and the medium containing non-adherent cells was removed and replaced with nutrient medium as described above. Supplemented medium was replaced every three days. On day 6, non-adherent and loosely adherent DCs were purified using MACS columns (Miltenyi Biotec, Bergisch Gladbach, Germany).

### 3.4. Flow Cytometric Analysis

Phenotypic maturation of DCs was analyzed by flow cytometry. Day 6 BMDCs were first incubated in the presence or absence of GP (100 μg/mL) for 48 h. Cells were then washed twice by 1% BSA-PBS. Cells were subsequently labeled with PE-CD11c, and FITC-CD80 or CD86 or MHC I-A/I-E antibodies (Biolegend, San Diego, CA, USA) for 30 min at 4 °C. Finally cells were washed twice with washing buffer and detected by flow cytometry (Becton Dickinson, San Jose, CA, USA). The obtained data were analyzed by the CellQuest software package (BD Biosciences, San Jose, CA, USA).

### 3.5. Endocytosis Assay

To analyze the endocytosis of DCs, day 6 BMDCs were first incubated in the presence or absence of GP for 48 h. Then, 2 × 10^5^ cells were incubated at 37 °C for 1 h with 1 mg/mL FITC-dextran (42,000 Da; Sigma-Aldrich, St. Louis, MO, USA). After incubation, cells were washed twice with cold washing buffer (HBSS) and FITC-dextran stained DCs were analyzed by flow cytometry. In the parallel experiment, 2 × 10^5^ DCs were incubated at 4 °C for 1 h.

### 3.6. Cytokine Assays

Levels of IL-12p70 and IFN-γ in culture supernatants were quantified using an enzyme-linked immunosorbent assay (ELISA) kit according to the manufacturer’s instructions (Genetimes Technology, Inc., Shanghai, China).

### 3.7. Mixed Lymphocyte Reaction (MLR)

CD3+ T cells from splenocytes of BALB/c mice were isolated using MACS columns (Miltenyi Biotec). Purity was greater than 96%. Day 6 BMDCs were incubated in the presence or absence of GP for 48 h, subsequently treated with 25 mg/mL mitomycin C (AppliChem, Darmstadt, Germany) for 45 min, and then washed twice with PBS. CD3+ T cells were added to each well and cultured for an additional 120 h at 37 °C. CD3+ T cell proliferation was determined using a WST-1 assay (Roche, Basel, Switzerland). Results are expressed as the mean of three wells from three individual experiments.

### 3.8. Real-Time PCR

Total RNA was isolated from DCs using a Trizol Reagent Kit (CWBIO Co., Ltd., Beijing, China), according to the manufacturer’s instructions. Single-strand cDNA was synthesized from 2 μg RNA using the HiFi-MMLV cDNA Kit (CWBIO Co., Ltd.). Real-time PCR was performed using the QIAGEN Rotor gene Q and Real SYBR Mixture Commercial Kits (CWBIO Co., Ltd.). GAPDH was used as a control. The primers used to detect IL-12 p40 were the forward primer 5'-TGTGCTCGTGGCCTGATC-3' and the reverse primer 5'-CTACGCAGCCCTGATTGA-3'. The primers used to detect GAPDH were the forward primer 5'-CTCATGACCACAGTCCATGC-3' and the reverse primer 5'-CACATTGGGGGTAGGAACAC-3'. The cycling profile for each run was 94 °C for 2 min and 40 cycles of 94 °C for 10min followed by 60 °C for 30 s. All samples were measured in triplicate. The relative mRNA levels of target genes were calculated by using the comparative 2^−^^△△Ct^ methods.

### 3.9. Neutralization Experiments

To examine the effect of GP on TLR4 and TLR4-downstream signal pathways, day 6 BMDCs were pre-treated with 20 µg/mL WTS510 (TLR4 neutralization antibody) (Biolegend), 20 µM PDTC (a specific blocker of NF-κB), 20 µM SB203580 (a specific blocker of p38 MAPK), 40 µM PD98059 (an inhibitor of the ERK pathway) or 20 µM JNK inhibitor (an inhibitor of the JNK pathway) (Beyotime Institute of Biotechnology, Haimen, Jiangsu, China) for 1 h and subsequently stimulated with GP (100 μg/mL) for 24 h. Isotype control mAb was added into control group. The cell culture supernatants were collected and IL-12 p70 was analyzed by ELISA.

### 3.10. Statistical Analysis

Data are presented as the mean ± SD. Differences were evaluated using Statistical Package for Social Science 11.0 (SPSS11.0, Chicago, IL, USA). Statistical analysis was performed using One-way ANOVA followed by least-significant difference (LSD). *p* < 0.05 was considered to be statistically significant.

## 4. Conclusions

In conclusion, we have demonstrated that GP can induce DC maturation. Additionally, we have determined that the mechanism by which GP exerts its effects may be through the regulation of the TLR4-related signaling pathway. Therefore, GP may be useful as part of the treatment regimen that regulates DC maturation.
